# Targeted Therapies in Epithelial Ovarian Cancer

**DOI:** 10.3390/cancers2010088

**Published:** 2010-02-23

**Authors:** Emma Dean, Loaie El-Helw, Jurjees Hasan

**Affiliations:** Christie Hospital NHS Foundation Trust / Wilmslow Road, Manchester, M20 4BX, United Kingdom; E-Mails: emma.dean@christie.nhs.uk (E.D.); loaie.elhelw@christie.nhs.uk (L.E.)

**Keywords:** ovarian cancer, targeted therapy

## Abstract

Molecularly targeted therapy is relatively new to ovarian cancer despite the unquestionable success with these agents in other solid tumours such as breast and colorectal cancer. Advanced ovarian cancer is chemosensitive and patients can survive several years on treatment. However chemotherapy diminishes in efficacy over time whilst toxicities persist. Newer biological agents that target explicit molecular pathways and lack specific chemotherapy toxicities such as myelosuppression offer the advantage of long-term therapy with a manageable toxicity profile enabling patients to enjoy a good quality of life. In this review we appraise the emerging data on novel targeted therapies in ovarian cancer. We discuss the role of these compounds in the front-line treatment of ovarian cancer and in relapsed disease; and describe how the development of predictive clinical, molecular and imaging biomarkers will define the role of biological agents in the treatment of ovarian cancer.

## 1. Introduction

Ovarian cancer is a leading cause of cancer mortality being the most frequent cause of death from gynaecological cancer, and the fourth most frequent cause of death from cancer in women in Europe and the USA. In 2002, there were an estimated 204,000 new cases of ovarian cancer and 125,000 deaths due to this disease worldwide. The incidence rates of ovarian cancer are highest in the Western world, where it is the leading cause of death from gynecological malignancies [1,2].

The 5 year survival rate of ovarian cancer ranges from 30% to 92%, depending on the stage at diagnosis [2]. Seventy-five percent of ovarian cancer patients present with evidence of metastatic spread beyond the ovaries and require combined surgery and chemotherapy [3]. The relapse rate in early stage ovarian cancer ranges between 10%–40% whilst more than 50% of patients with advanced disease will eventually progress. Only 10%–30% of such patients attain long term survival with a median progression-free survival of 18 months [4,5,6,7,8].

Frontline treatment with a platinum-paclitaxel combination is the internationally accepted standard of care in chemo-naïve advanced or recurrent ovarian cancer. At relapse, platinum compounds remain the mainstay of treatment. In platinum-sensitive disease i.e. where the treatment-free interval (TFI) is more than 6 months the response rates can be greater than 50%, but it is only 10–20% for platinum-resistant disease (TFI <6 months) and less for platinum-refractory disease where the disease progresses on treatment. The latter are therefore usually treated with other non-platinum agents, such as liposomal doxorubicin, gemcitabine, topotecan, etoposide and hormonal therapies [9].

A greater understanding of tumour biology and molecular pathways that mediate cancer progression and drug resistance has led to the development of various molecular targeted therapies such as monoclonal antibodies, small molecule receptor tyrosine kinase inhibitors and agents blocking downstream signalling pathways. The development of molecular-directed therapies received a tremendous boost with the confirmation of efficacy of imatinib in GISTs [10], of bevacizumab in the first-line treatment of metastatic colorectal cancer [11,12] and other solid tumours such as breast [13] and lung [14], of sorafenib in liver [15] and renal cell carcinomas [16], and also of sunitinib in renal cell carcinomas [17]. This review focuses on the recent developments with new biological agents in the treatment of advanced and recurrent ovarian carcinoma.

## 2. Antiangiogenic Therapy in Ovarian Cancer

Angiogenesis is essential for normal ovarian physiological processes such as follicle maturation and the development of corpus luteum during normal ovulation. Pathological neoangiogenesis on the other hand is a key factor in the growth and development of ovarian tumours. Epithelial ovarian cancer over-expresses vascular endothelial growth factor (VEGF) and other proangiogenic proteins like platelet-derived growth factor (PDGF) and angiopoietin. Increased microvessel density and over-expression of VEGF and its receptor have been correlated with aggressive clinical features, formation of ascites and poor outcome. Conversely, in animal models it has been shown that VEGF blockade inhibits ascites formation and slows ovarian cancer growth [18,19,20,21,22].

Antiangiogenic therapies inhibit new blood vessel growth, induce endothelial cell apoptosis, block the incorporation of haematopoietic and endothelial progenitor cells into new blood vessels, and normalize the vasculature [23]. These effects are mediated by the binding of VEGF to the vascular endothelial growth factor receptors (VEGFR), inhibiting receptor tyrosine kinase activation and downstream molecules, or occluding fragile tumour vasculature by vascular disrupting agents [24,25]. Due to its central role in tumour angiogenesis, the VEGF/VEGFR axis has been identified as a prime target in novel anticancer drug development.

### 2.1. Bevacizumab (Avastin)

Bevacizumab (Avastin, Genentech) is a 149-kDa recombinant humanized monoclonal IgG1 antibody directed against human VEGF. In preclinical models of ovarian cancer, a VEGF-targeted antibody alone had minimal effect on tumour burden, but markedly decreased ascites [26]. However, in combination with paclitaxel, tumour burden along with ascites was significantly decreased [27]. Similarly, in an ovarian cancer xenograft these results were reproduced in combination with cisplatin, and continuation of bevacizumab after induction chemotherapy significantly delayed recurrence and prolonged survival suggesting a role as maintenance therapy [28]. Bevacizumab has been evaluated as a single agent as well as in combination with chemotherapy in patients with ovarian cancer in a number of clinical trials, summarized in Table 1 [29,30,31,32,33,34].

**Table 1:** Phase II trials of antiangiogenic agents in ovarian cancer.

Regimen	Dose	Patient Number	Platinum sensitivity (%)	Response by RECIST (%)		Median Survival (months)	
				Partial Response	Stable Disease	PFS	OS
Bevacizumab Cannistra *et al.*, 2007[29]	15 mg/kg q3w	44	0	16	61	4.4	10.7
Bevacizumab Burger *et al.*, 2007 [32]	15 mg/kg q3w	62	58	21 (CRR)	52	4.7	17
Bevacizumab + cyclophosphamide Garcia *et al.*, 2008 [33]	10 mg/kg q2w 50 mg/d	70	60	24	63	7.2	16.9
Bevacizumab + Cyclophosphamide Chura *et al.*, 2007 [39]	10 mg/kg q2w 50 mg/d	15	0	53 (13 CRR)	20	3.9	NR
Bevacizumab + carboplatin + paclitaxel Micha *et al.*, 2007 [40]	15 mg/kg q3w AUC 5 q3w 175 mg/m^2^ q3w	20	First line	80 (CRR)	5	NR	NR
Bevacizumab + carboplatin + paclitaxel Campos *et al.*, 2007 [41]	15 mg/kg q3w AUC 5 q3w 175 mg/m^2^ q3w	58	First line	75	NR	11	NR
Aflibercept Tew *et al.*, 2007 [43]	2 or 4 mg/kg q2w	162	0	11	NR	NR	NR

RECIST (Response Evaluation Criteria in Solid Tumors); PFS: progression free survival; OS: overall survival; NR: not reported; CRR: complete response rate.

#### 2.1.1. Single Agent Bevacizumab in Patients with Recurrent Ovarian Cancer

Single agent bevacizumab has been evaluated in the treatment of patients with recurrent ovarian carcinoma in two phase II clinical trials at a dose of 15 mg/kg administered every 3 weeks in patients who had received one to three previous chemotherapeutic regimens [29,32], Table 1. Observed response rates were 16%–21% with a median progression free survival of 4.4 and 4.7 months respectively. Clinical activity was independent of platinum sensitivity (the Cannistra study only recruited patients with platinum-resistant disease [29], while the Burger study was open to patients with both platinum-resistant and platinum-sensitive disease [32]). These results are notable because, apart from renal carcinoma, ovarian cancer is the only epithelial tumour type in which single agent bevacizumab has been shown to produce clinically significant activity. 

#### 2.1.2. Bevacizumab in Combination with Chemotherapy in Patients with Recurrent Ovarian Cancer

Cytotoxic chemotherapy is conventionally administered at a dose close to its maximum tolerated dose, with breaks to allow the recovery of normal tissues. Chemotherapy at this dose does kill proliferating vascular endothelial cells, but recovery happens rapidly. In animal models, lower doses of chemotherapy for example, paclitaxel, vinorelbine and cyclophosphamide given frequently (metronomic dosing) target proliferating endothelial cells leading to their apoptosis with few side effects and no significant direct tumour cytotoxicity [35,36,37]. 

Metronomic docetaxel chemotherapy in combination with AEE788, (a combined EGFR and VEGFR inhibitor), has shown encouraging activity in an orthotopic mouse model of ovarian cancer utilising a cell line resistant to conventional chemotherapy dosing [38]. This approach has also been explored in two phase II clinical trials of bevacizumab (10 mg/kg, every 2 weeks) and metronomic oral cyclophosphamide (50 mg daily) [33,39], Table 1. Promising clinical activity was demonstrated in both trials and in the study by Chura *et al.* [39] a response rate of 53% was observed (two patients had a complete response, six patients a partial response) in a heavily pretreated population (median number of previous chemotherapy regimens 8). 

A multi-institutional retrospective evaluation of factors predictive of toxicity and efficacy of bevacizumab in patients with recurrent ovarian cancer was performed by Wright *et al.* [34]. Single-agent bevacizumab was administered to 12 patients, while 50 received the drug in combination with cytotoxic agents. The addition of a cytotoxic agent to bevacizumab improved objective response rates (43% *vs.* 10%, p = 0.07) at the cost of increased toxicity. Grade 3–5 toxicities occurred in 15 (24%) patients, including hypertension (7%), gastrointestinal perforations (7%), and chylous ascites (5%). Gastrointestinal perforations occurred in heavily pretreated patients who were responding to therapy. Development of chylous ascites and gastrointestinal perforations appeared to correlate with tumor response. The overall response rate was 36%, with stable disease in 40%. The GOG 213 study aims to recruit 1,600 patients in a bifactorial design of carboplatin and paclitaxel alone or in combination with bevacizumab followed by bevacizumab and secondary cytoreductive surgery in platinum-sensitive, recurrent ovarian, fallopian tube and primary peritoneal carcinoma.

#### 2.1.3. Bevacizumab combination with Carboplatin and Paclitaxel in the first-line setting

In chemotherapy-naïve patients with advanced ovarian carcinoma, carboplatin/paclitaxel and bevacizumab produced a response rate of 75%–80% with acceptable toxicity [40,41], Table 1. Two large randomised Phase III trials are evaluating the benefit of adding bevacizumab to carboplatin and paclitaxel in the first-line setting: GOG 218 and ICON7. The primary objective of GOG 218 is to evaluate the impact on overall survival of 5 concurrent cycles of bevacizumab (15 mg/kg q3w) with 6 cycles of carboplatin and paclitaxel, when compared with 6 cycles of carboplatin and paclitaxel in women with newly diagnosed stage III and IV epithelial ovarian and peritoneal primary cancer. Bevacizumab is omitted with the first cycle of chemotherapy in order to reduce the risk of wound-healing complications. This study also evaluates if maintenance bevacizumab for 16 cycles beyond the 6 cycles of standard carboplatin and paclitaxel improves survival when compared with 6 cycles of carboplatin and paclitaxel. ICON7 has a similar design, without the placebo maintenance arm and with bevacizumab at a dose of 7.5 mg/kg q3w. It also includes patients with high risk early stage ovarian cancer (FIGO stage I clear cell or grade 3) as well as advanced stage patients. The different dosing schedules in these trials will hopefully address the question of whether a 5 mg/kg/week dose level is appropriate (utilized in most ovarian cancer studies, Table 1), or whether a lower dose is just as effective. At present, there does not appear to be any evidence to suggest a dose response effect with bevacizumab in ovarian cancer. This correlates with a phase II trial in metastatic colorectal cancer which used two dosing schedules of bevacizumab (5 mg/kg q2w and 10 mg/kg 2qw) with fluorouracil/leucovorin, and showed no significantly significant difference in overall survival between the two dosing schedules compared with control [42]. Bevacizumab regimens in other solid tumours e.g. lung, breast, and renal cancer have also employed varying dosing regimens.

### 2.2. Other anti-VEGF Agents

#### 2.2.1. Aflibercept

One of the most effective ways to block the VEGF signalling pathway is to prevent VEGF from binding to its normal receptors by administering decoy VEGF receptors. Aflibercept (VEGF Trap), is one such soluble decoy receptor. It is a fusion protein containing the VEGF-binding domains of both VEGFR-1 and -2 linked through the Fc region of human IgG1 and is a potent inhibitor of VEGFA. In a Phase II double-blind study of patients with platinum-resistant ovarian cancer, 162 patients were treated at two dose levels of 2 mg/kg or 4 mg/kg every 2 weeks. Independently assessed response rates were 11%. Side-effects were reassuringly typical of this class of drugs with hypertension being the most common [43]. However given the modest response rate, it is unlikely single agent aflibercept will have major role in the treatment of ovarian cancer.

#### 2.2.2. Receptor Tyrosine Kinase Inhibitors

Activated receptor tyrosine kinases (TKIs) phosphorylate numerous signalling molecules activating downstream signal transduction pathways leading to tumour cell proliferation and survival. These phosphorylation dependent mechanisms are essential for promoting the activity of growth factors like VEGF and PDGF. Blocking phosphorylation by targeting the intracellular component of tyrosine kinase thereby inhibiting the biological activity of VEGF is therefore an effective antitumour strategy.

Promising results from several phase II trials investigating single agent small molecule TKIs, that target VEGFR in relapsed ovarian cancer are summarized in Table 2. These include sunitinib, cediranib, sorafenib, pazopanib, and imatinib [44,45,46,47,48,49,50]. The experience with imatinib, a PDGFR and c-Kit inhibitor, was however disappointing with minimal demonstrable activity as a single agent [45,46]. This may reflect redundancies in signalling pathways and activation of other mechanisms like the Akt pathway. With other compounds, objective response rates of 10%–15% have been observed. Most toxicities are dose-dependent; tiredness, diarrhoea and lethargy being common (Table 3). Given the convenience of oral administration, it is likely these drugs will have a key role as maintenance therapy in advanced ovarian cancer. The development of effusions in patients on Sunitinib during a planned two-week treatment break is likely due to sudden release of VEGF inhibition, and also supports a continuous dosing strategy [44].

**Table 2:** Phase II trials of oral VEGFR tyrosine kinase inhibitors in relapsed ovarian cancer.

Regimen	Dose	Patient Number	Platinum Resistant (%)	Efficacy (%) (CR+PR+SD)
Sunitinib Biagi *et al.*, 2008 [44]	50 mg o.d. 28 days, 6-weekly cycle	17	NR	71
Cediranib Matulonis *et al.*, 2008 [45]	45 mg o.d. reduced to 30 mg o.d. (n=18)	29 (27 evaluable)	57	30
Sorafenib Matei *et al.*, 2008 [46]	400 mg b.i.d.	73 (59 evaluable)	NR	37
Pazopanib Friedlander *et al.*, 2007 [47]	800 mg o.d.	17 (15 evaluable)	26	27 47 (CA-125 response)
Imatinib Coleman *et al.*, 2006 [48]	600 mg o.d.	28	100	33
Imatinib Posadas *et al.*, 2007 [49]	400 mg b.i.d. 16 patients dosed at 600 mg o.d	23	NS	9
Cediranib Hirte *et al.*, 2008 [50]	45 mg o.d. reduced to 30 mg o.d. (n=8) and 20 mg o.d. (n=8)	60	57	70

NR: Not reported; o.d. once a day; b.i.d. twice a day; PR: partial response; PS: performance status.

**Table 3:** Toxicity profile of antiangiogenic agents.

Regimen	Reference	Toxicities (CTCAE)
Bevacizumab	Cannistra *et al.*, 2007 [29]	16% proteinuria, 11% GIP, 9% HT, 7% ATE, 5% pain, 5% fatigue
Bevacizumab	Burger *et al.*, 2007 [32]	10% HT, 0% GIP
Bevacizumab + cyclophosphamide	Garcia *et al.*, 2008 [33]	11% HT, 16% proteinurea, 6% GIP
Bevacizumab + carboplatin + paclitaxel (first line)	Micha *et al.*, 2007 [40]	48% NP, 10% HT, 10% VTE (1 prior to bevacizumab, 1 with portacath), 10% neuropathy
Bevacizumab + carboplatin + paclitaxel	Campos *et al* 2007 [51]	22% NP, 16% VTE, 4% HT, 8% pain, 3% GIP
Aflibercept	Tew *et al* 2007 [43]	18% HT, 1% GIP
Cediranib	Matulonis *et al.*, 2008 [45]	HT (45%), fatigue (17%), diarrhoea (10%)
Imatinib	Coleman *et al.*, 2006 [48]	Fatigue (17%), Nausea and vomiting (7%) Ascites (7%)
Imatinib	Posadas *et al.*, 2007 [49]	26% ascites, 17% pleural effusion, 13% fatigue, 13% cytopenia
Cediranib	Hirte *et al.*, 2008 [50]	HT(33%), fatigue (20%)
Sunitinib	Biagi *et al.*, 2008 [44]	Fatigue, hand – foot syndrome, neutropenia, Thrombocytopenia, pleural effusion.
Sorafenib	Matei *et al.*, 2008 [46]	Rash (17%), metabolic, (15%), gastrointestinal (4%)
Pazopanib	Friedlander *et al.*, 2007 [47]	Diarrhoea (12%), ALT elevation (12%)
Bevacizumab + sorafenib	Azad *et al.*, 2008 [51]	26% HT, 8% GIP, 5% proteinurea, 11% LFT abnormality

CTCAE, Common Toxicity Criteria of Adverse Events; HFS: hand–foot syndrome; GIP: gastrointestinal perforation; HT: hypertension; ATE: arterial-thrombotic events; LFT: liver function abnormality; NP: neutropenia; VTE: venous thrombo-embolism events; NV: nausea or vomiting.

### 2.3. Combination Anti-VEGF and Multi-target Therapy

Vertical blockade by inhibition at different points in the VEGF signalling pathway has been mooted as a strategy to enhance efficacy. This was explored in a phase I study of sorafenib and bevacizumab which demonstrated durable partial disease responses in 6 of 13 ovarian cancer patients recruited. Unexpectedly, toxicity was substantially higher than that with single-agent anti-VEGF therapy, with two thirds of the patients developing hypertension and 79% incidence of grade 1–3 hand–foot syndrome. Enteral fistulae were also seen in 2 of the 13 ovarian cancer patients in the study [51]. 

EGFR and VEGF share common downstream signalling pathways. VEGF is down-regulated by EGFR inhibition, and conversely blockade of VEGF may also inhibit EGFR autocrine signalling [52]. Dual blockade of these two molecular targets was therefore hypothesized to produce a synergistic effect. However two fatal bowel perforations amongst thirteen recruited patients associated with a higher than expected incidence of grade 3 diarrhoea lead to the premature termination of a phase II study of combination bevacizumab and erlotinib in patients with recurrent ovarian cancer [53]. Poorer outcomes and increased toxicity with combination biological therapy have also been reported in other solid tumours and led to the early termination of a front-line study with bevacizumab and panitumumab in metastatic colorectal cancer [54]. An awareness of the likelihood of increased toxicity with new combinations of novel agents is necessary going forward with the next generation of clinical trials with molecularly targeted agents. Table 3 outlines the different side effects observed for several different antiangiogenic agents and combination therapies.

An approach that combines bevacizumab and a Vascular Disrupting Agents (VDA) was explored as preclinical data provide evidence for synergy between a VDA, which induces a surge in VEGF-stimulated circulating endothelial progenitor cells, and bevacizumab, which suppresses this VDA-induced effect. No additive toxicity was seen in phase I study of bevacizumab and a VDA combretastatin 4A phosphate [55,56].

### 2.4. Vascular Disrupting Agents

Vascular disrupting agents (VDAs) target endothelial cells and pericytes of the already established tumour vasculature, resulting in tumour ischemia and necrosis. VDAs have been divided into two types: ligand-directed VDAs and small molecules. Ligand-directed VDAs consist of targeting and effector moieties that are linked together. Their clinical efficacy appears limited because of cost and a lack of specificity and toxicity. Small molecules include two classes: the synthetic flavonoids, which work through induction of local cytokine production, and the tubulin-binding agents [57]. Combretastatin, a tubulin-binding agent has shown synergistic activity when administered in combination with chemotherapy in ovarian cancer xenografts. A phase II study of combretastatin in combination with carboplatin and paclitaxel was reported at the American Society for Clinical Oncology 2009 meeting [58]. Toxicity was manageable, notably cardiac toxicity was minimal and hypertension was easily controlled on medication. The modest response rate – 14% however suggests this combination is unlikely to progress further.

### 2.5. Safety Profile of Antiangiogenic Agents

Bevacizumab is associated with several well recognized toxicities including hypertension, proteinuria and minor bleeding. More serious, but rarer events, are arterial thrombosis, impaired wound healing, major bleeding, gastrointestinal perforation (GIP), reversible posterior leukoencephalopathy and tracheo-oesophageal fistulae. A metaanalysis of all prospective randomized studies of bevacizumab in solid tumors showed the risk of GIP to be 0.9% with a mortality of 21.7% [59]. Incidence in ovarian cancer appears to be higher with combined data from several nonrandomised series putting the risk of GIP with bevacizumab in ovarian cancer at 5.4% (nearly twice the risk in colorectal cancer 3.1%), [60]. However, GIP risk in individual studies varies from 0–15% and does not appear to be a dose-related effect or related to the addition of chemotherapy. The aetiology of GIP with bevacizumab in ovarian cancer remains obscure, but advanced disease is associated with diffuse peritoneal and serosal bowel involvement impairing bowel motility and blood supply. Additionally, chronic subacute bowel obstruction and poor nutrition predisposes patients to higher risk of GIP. The clear inference is that bevacizumab is not best suited for ovarian cancer patients with extensive serosal disease and patients at risk of bowel obstruction.

Hypertension is a side-effect noted with bevacizumab and other small molecule TKIs. A metaanalysis of randomized controlled trials with patients receiving bevacizumab showed a relative risk of 3.0 for hypertension at low doses (2.5–7.5 mg/kg) and 7.5 for high doses (10–15 mg/kg); and for proteinuria 1.4 at low doses and 1.6 for higher doses [61]. There is no consistent correlation between duration of bevacizumab treatment and development of hypertension, with the median time to development of hypertension 131 days for bevacizumab [62] and days to weeks for oral TKIs [63]. In most cases, blood pressure returns to baseline following discontinuation of therapy. Oral TKIs have been noted to cause gastrointestinal effects, principally diarrhoea. Rarely, hypothyroidism has been observed. Table 3 outlines the different side effects observed for several different antiangiogenic agents.

## 3. Epidermal Growth Factor Receptor

The epidermal growth factor receptor (EGFR, also named ErbB1) is a tyrosine kinase receptor of the ErbB family (ErbB1-4) that is abnormally activated in many epithelial tumours. Receptor activation leads to recruitment and phosphorylation of several downstream intracellular substrates, leading to mitogenic signalling and other tumour-promoting cellular activities. In human tumours, receptor overexpression correlates with a more aggressive clinical course. Taken together, these observations indicate that the EGFR is a promising target for cancer therapy. The two main classes of compounds specifically targeting EGFR include low-molecular weight Tyrosine Kinase Inhibitors (TKIs) and anti-EGFR monoclonal antibodies (MAbs) and these are at different stages of development, summarized in Table 4. 

**Table 4:** Anti-EGFR Monoclonal Antibodies and low-molecular weight Tyrosine Kinase Inhibitors (TKIs) in patients with ovarian cancer.

Reference	Regimen	Patient Number	Phase	RR (%)	SD (%)	PFS (mo)	Side effects- G3/4 toxicity
**A: Anti-EGFR Monoclonal Antibodies: Anti-Human Epidermal Growth Factor Receptor 2 (Her-2)/neu**							
Bookman *et al.*, 2003 [75]	Trastuzumab (4 mg/kg loading, 2 mg/kg q7d)	41	II	7.3	39	2	Gastrointestinal, 6%; neuropathy, 9% and fatigue, 8%
**B. Anti-EGFR Monoclonal Antibodies: Anti-Human Epidermal Growth Factor Receptor 1 (anti ErbB1/EGFR/HER1)**							
Agus *et al.*, 2005 [119]	Pertuzumab (0.5–15 mg/kg q3w)	21 (3 ovarian cancer)	I	10 (33)	29 (33)	NS (10)	Abdominal pain 14%, dyspnoea 10% , vomiting 5%, nausea 5%, diarrhoea 5%
Seiden *et al.*, 2007 [68]	Matuzumab/EMD 72000 800 mg q7d	37	II	0	16	54	Nausea 6%, headache 3%, abdominal pain 3%, diarrhoea 3%, vomiting 3%, myalgia 3%, acute pancreatitis 3%, intestinal obstruction 3%,
Aghajanian *et al.*, 2005 [71]	Cetuximab 400 mg/m2 loading then 250 mg/m^2^ q7d (+ paclitaxel 175 mg/m^2^ and carboplatin AUC6 q3w)	17 Chemo-naïve, stage III-IV	II	87 (uCR)		NR	Febrile neutropenia (12%), diarrhea (6%), and hypersensitivity (6%)
**C. Small molecule EGFR TKIs**							
Finkler *et al.*, 2001 [70]	Erlotinib 150 mg o.d.	34 refractory to taxane- and/or platinum-therapy	II	9	44 (U)	NR	Acneiform rash 88%, diarrhea 4%.
Vasey *et al.*, 2004 [120]	Erlotinib 50–150 mg o.d. (+docetaxel 75 mg/m^2^ and carboplatin AUC5 q3w)	39 with surgical cytoreduction but chemo-naïve, 18 evaluable	Ib	61 (18 evaluable patients)	NR	NR	Skin rash 33%, diarrhea 8%, Plantar-palmer erythro-dysesthesia (PPE) 8%
Blank *et al.*, 2006 [121]	Erlotinib 150 mg o.d. (paclitaxel 175 mg/m^2^ and carboplatin AUC6 q3w)	47 (29 optimal cytoreduction [Op], 18 sub-optimal [S]), all chemo-naïve	II	53 had pCR in the Op group, 28had good response in the S group	NR	NR	skin rash (grade was not reported)
Slomovitz *et al.*, 2006 [122]	Gefitinib 250 mg o.d.(+ topotecan 2–4 mg/m2 d1, 8, 15 q28d)	13 (measurable disease after platinum + paclitaxel)	I	0 (11 evaluable patients)	36	NR	Thrombocytopenia 17%
Hariprasad *et al.*, 2006 [123]	Gefitinib 250 mg o.d.	32 advanced and recurrent epithelial ovarian carcinoma	II	NR	NR	56 (at 6 months)	Skin rash 16 %,
Mavroudis *et al.*, 2004 [124]	Gefitinib 250 mg o.d.(+ vinorelbine 20–25 mg/m^2^ and oxaliplatin 40–50 mg/m^2^)	33 recurrent or refractory ovarian cancer	I/II	48	NR	4.1 (CDDP-sensitive 8.6 (CDDP-resistant)	Neutropenia 48%, febrile neutropenia 12%, anemia 3%, diarrhea 9%, neurotoxicity 3%, rash 3% and transaminitis 3%.
Krasner *et al.*, 2005 [125]	Anastrazole 1 mg o.d.and gefitinib 250 mg o.d.	35, asymptomatic recurrent mullerian cancer	II	4 (CR), (23 evaluable patients)	61	NR	Rash 3%, diarrhea 3%
Minami *et al.*, 2004 [126]	Lapatinib 900–1800 mg/day Dual ErbB1 (EGFR) and ErbB2 (HER2) TKI	24, patients with solid tumours including cervical and ovarian cancers	I	8	50	NR	Diarrhea 33%, elevation of GGT 33%.%
Campos *et al.*, 2007 [127]	Canertinib 50 or 200 mg o.d for 21 days q28d Pan-ErbB TKI	105 Platinum resistant patients	II	0	28 (50 mg) 34 (200 mg)	0 and 9 (1-year PFS in 50 and 200 mg dose respectively)	G2/3 toxicities: diarrhea 85%, stomatitis 69%, rash 58%.

RR: response rate, SD: stable disease, CDDP: cisplatin; PFS: progression free survival; mo: months; U: Unconfirmed; uCR: unconfirmed complete response; CR: complete response; PR: partial response. NR: not reported; o.d. once daily; GGT: Gamma glutamyl transpeptidase

EGFR is overexpressed in a high proportion of ovarian carcinomas [64,65]. Following positive results in human xenograft models which showed potentiation of cytotoxicity when anti-EGFR agents were administered with chemotherapy [66], several studies investigated monoclonal antibodies such as cetuximab and matuzumab and oral receptor tyrosine kinase inhibitors of EGFR like gefitinib and erlotinib in ovarian cancer [67,68,69]. In relapsed platinum-resistant ovarian cancer single agent therapy was associated with modest response rates of under 10% [70] although higher response rates were noted when combined with chemotherapy in platinum-sensitive patients and in first-line therapy [71]. Common toxicities observed include fatigue, skin rash and GI toxicities, Table 4. A GCIG intergroup study of erlotinib maintenance versus observation in patients with high risk stage I and stage II–IV epithelial ovarian cancer completed recruitment in 2008 and results are awaited.

Biomarker driven anti-EGFR therapy is now established in metastatic colorectal cancer with the discovery that tumours with mutated KRAS do not respond to anti-EGFR therapy [72]. This, as yet, has not been explored with anti-EGFR therapy in ovarian cancers although the KRAS pathway is implicated in ovarian tumorigenesis particularly in low grade serous and mucinous carcinomas [73]. Interestingly, subset analysis of ovarian cancer patients treated with gefitinib suggests the likelihood of response to be related to the presence of a mutation in the catalytic domain of EGFR as previously described in lung cancer. However the mutation appears to be a rare event being found in only 3% of the study population [74]. Likewise ErbB2 (also named HER2) overexpression is found in only a minority of ovarian carcinomas (5%). There is nonetheless no correlation between ErbB2 overexpression and response to trastuzumab, an ErbB2 monoclonal antibody. In a study of 837 patients with recurrent or chemorefractory ovarian cancer treated with trastuzumab, a response rate of 7% was obtained in 47 patients with ErbB2 overexpressing tumours [75]. Pertuzumab, another humanised monoclonal antibody that inhibits dimerization of ErbB2 with other EGFRs has shown unexceptional activity in recurrent ovarian carcinoma with response rates under 5% when administered as a single agent [76]. Nevertheless a post hoc analysis of the results of a completed randomized Phase II trial of gemcitabine with or without pertuzumab in patients with platinum-resistant ovarian cancer suggests that low ErB3 levels (also named HER3) may mark a group of women who may benefit from the addition of pertuzumab to chemotherapy [77].

## 4. PARP Inhibitors

Poly (ADP-ribose) polymerase (PARP) is a key enzyme involved in the surveillance and maintenance of genomic integrity (reviewed in [78]). It functions as both a molecular sensor of DNA damage (resulting from interruption of the sugar-phosphate backbone or from base injury) and following its detection, efficiently recruits partner proteins to repair damage by the single-strand break repair (SSBR) and base excision repair (BER) pathways [79]. PARP inhibition results in the accumulation of DNA single-strand breaks which convert into DNA double-strand breaks (DSBs) In normal cells, DSBs are effectively repaired. However, tumour cells that are deficient in the tumour suppressors BRCA1 and BRCA2 proteins, which are important for efficient repair of DSBs by homologous recombination repair, use alternative DNA repair pathways such as base excision repair to compensate for nonfunctional homologous recombination [80,81]. Therefore, PARP inhibitors may selectively kill tumour cells, exploiting the concept of synthetic lethality by combining base excision repair inhibition with defective homologous recombination DNA repair pathway. In fact, PARP inhibition can also sensitise non-neoplastic BRCA2-deficient cells, potentially allowing use as a prophylactic treatment in women that are heterozygous for the BRCA2 gene [82]. 

Olaparib (AZD2281, KU-0059436; AstraZeneca) is an potent, orally active, small-molecule PARP inhibitor which inhibits PARP1 and PARP2 with a mean IC50 of 2 nM [83]. Preclinical studies confirmed that olaparib induces synthetic lethality in homologous recombination repair defective cells, including BRCA-deficient tumours [81]. BRCA-deficient cells were up to 1000-fold more sensitive than wild-type cells to PARP inhibition. Conversely, cells heterozygous for BRCA mutations with an intact homologous recombination function exhibited a similar lack of sensitivity to PARP inhibitors as wild-type cells. Collectively, this data suggests that a therapeutic index for anti-tumour therapy may exist in BRCA-associated ovarian cancer, with preferential lethality of tumour cells [81,84].

The Phase I trial of olaparib was inclusive of patients with a wide range of drug-resistant cancers for the escalation phase [85]. After the maximum tolerated dose (MTD) was defined, the dose expansion phase was enriched with patients carrying BRCA1 or BRCA2-deficient ovarian tumours. Olaparib was well tolerated with mainly grade 1–2 gastrointestinal toxicities of nausea, vomiting, dysgeusia (loss of taste), anorexia, diarrhoea and constipation. Other toxicities included fatigue and one patient had a dose-limiting neurocognitive toxicity. Little myelosuppression was observed. Pharmacokinetics were dose-proportional up to 200 mg bd and pharmacodynamic studies showed significant PARP1 inhibition in both tumour tissues and surrogate tissues (mainly peripheral blood mononuclear cells) at a dose levels of 100mg twice daily and higher. The MTD was identified as 400 mg bd. Thirty-two patients with BRCA-deficient ovarian cancer (the majority of whom were platinum resistant / refractory) were evaluable for response. Compelling anti-tumour activity was seen in all patient groups receiving doses of 100 mg bd and above: fourteen partial responses (thirteen fulfilling GCIG-Ca125 criteria and ten RECIST criteria) and eight stable diseases.

The results of the Phase II trial, presented at ASCO 2009, primarily aimed to test the efficacy of olaparib in confirmed BRCA1/BRCA2 carriers with advanced chemotherapy-refractory ovarian cancer [86]. In this single-arm study, two sequential cohorts received continuous oral olaparib in 28-day cycles; at the biologically effective dose of PARP inhibition of 100 mg bd and at the MTD of 400 mg bd. At the time of interim analysis, fifty-seven patients had been enrolled (thirty-three patients at 400 mg bd and twenty-four patients at 100 mg bd), thirty-nine were BRCA1 deficient and eighteen BRCA2 deficient. The primary endpoint was objective response rate, which was reported as 33% and 13% for the 400 mg bd and 100mg bd cohorts respectively, and the clinical benefit rate (ORR and/or confirmed ≥50% decline in Ca125) was 58% and 17% respectively. Toxicity was mild and similar to that reported in the Phase I trial: grade 1/2 nausea (44%), fatigue (35%), and anaemia (14%). Grade 3 toxicity occurred infrequently, and comprised primarily nausea (7%) and leucopenia (5%). Importantly, toxicities in BRCA1/2 carriers were similar to that previously reported in noncarriers. Olaparib is also being evaluated in a randomized Phase II trial in comparison with pegylated liposomal doxorubicin in patients with BRCA-mutated ovarian cancer with a platinum-free interval of 0–12 months. Other PARP inhibitors currently undergoing clinical development in BRCA-associated carriers with cancer are BSI-201 (BiPar Sciences), ABT-888 (Enzo), AG014699 (Pfizer) and MK4827 (MERCK).

PARP inhibitors may also have a role in sporadic ovarian cancers with homologous recombination defects, which might result from nonfunctional proteins rendering these cells sensitive to PARP inhibition [84]. These sporadic tumours behave like BRCA1 and BRCA2 deficient tumours but do not possess germ line mutations in either gene. Despite the genetic disparity, the similarities in behaviour between these sporadic tumours and BRCA1 and BRCA2 deficient tumours has been termed ‘BRCAness’ [87]. One molecular characterization study of forty-nine ovarian cancers suggested that approximately 50% of patients with high-grade serous or undifferentiated ovarian cancer had loss of BRCA function, [88]. It was also possible to subclassify high grade serous carcinomas into three groups: BRCA1 loss (genetic), BRCA1 loss (epigenetic), and no BRCA1 loss based on molecular alterations, which potentially may be used to guide treatment and determine prognosis. Olaparib is also being evaluated in this setting in a randomized placebo-controlled trial of maintenance therapy in patients with serous (sporadic) ovarian cancer at high risk of early recurrence. However, the future of personalised medicine in patients affected by ovarian cancer is dependent on the development of a robust qualified assay for detecting homologous recombination-defective tumours and the mechanism of BRCA1/2 loss. By this method patient populations can be selected molecularly, increasing the likelihood of clinical benefit from this targeted strategy.

## 5. PI3K-PTEN-Akt-mTOR Pathway

The PI3K-PTEN-Akt-mTOR pathway is implicated in multiple biological processes including cell proliferation, protein translation, autophagy, cell metabolism, cell proliferation, angiogenesis and prevention of apoptosis. Alterations of this signalling pathway are well documented in cancer and are believed to result in activation of downstream signalling pathways that drive oncogenesis [89]. For this reason, core components of the pathway are being targeted for anticancer therapy, and molecular markers identified that may be prognostic or predictive of responsiveness to therapy. In addition, the PI3K-PTEN-Akt-mTOR pathway mediates resistance to anticancer therapies. Combinations of inhibitors targeting this pathway with conventional cytotoxics, octreotide, trastuzumab or hormone therapy have been shown to be synergistic preclinically and in early clinical trials (reviewed in [90]).

### 5.1. PI3K-PTEN-Akt-mTOR Aberrations in Ovarian Cancer

PI3K has at least eight isoforms of catalytic subunit and seven regulatory subunits. The PI3K proteins implicated in tumorigenesis belong to a subclass called 1A. Genetic amplification of PIK3CA, which encodes p110α catalytic subunit of PI3K (Class IA catalytic subunit) have been observed in approximately 40% of ovarian cancers [91]. Transforming mutations and amplification result in increased PI3K activity, activation of Akt signalling and oncogenesis. PI3KCA mutations have been associated with endometrioid and clear cell ovarian histological subtypes [92], while PIK3CA amplification is observed in all histological subtypes. 

PI3K signalling is negatively regulated by the protein phosphatase PTEN (phosphatase and tensin homolog deleted on chromosome ten). PTEN can be considered as a tumour suppressor, preventing Akt activation through the dephosphorylation of a PI3K lipid signalling product. The elimination of PTEN activity (by mutation, deletion or epigenetic silencing) can lead to oncogenesis through activation of Akt, which in turn upregulates mTOR activity. Therefore, cancer cells deficient in PTEN are an attractive target for mTOR inhibitors. PTEN aberrations have been implicated in ovarian cancers, especially those with an endometrioid histology [93], and loss of functional PTEN occurs in 40%–80% of patients with endometrial cancer [94].

Akt is an intracellular serine/threonine kinase which is a critical regulatory switch, and is believed to phosphorylate over 9,000 proteins (reviewed in [95]). Consequently, it has diverse roles in the cancer cell: tumour invasion, metastasis, cell cycle regulation, prevention of apoptosis and pro-angiogenesis. Three Akt isoforms have been identified, with Akt1 and Akt2 most commonly associated with ovarian cancer [96]. In addition to Akt activation as a consequence of PI3K or PTEN aberrations, VEGFR-A/VEGFR-2 triggers the antiapoptotic PI3K-PTEN-Akt-mTOR cascade. Therefore, inhibition of VEGF-A is both antiangiogenic and proapoptotic [97]. The anti-apoptotic role of Akt has also been shown to mediate chemotherapy resistance to both taxanes and cisplatin chemotherapy [98,99], by its interaction with survivin (an inhibitor of apoptosis protein) and by blocking p53 response to apoptotic stimuli respectively. Conversely, the PI3K inhibitor LY294002 (which prevents phosphorylation and activation of Akt) enhanced paclitaxel-induced apoptosis in vitro and in vivo in ovarian cancer [27].

Clinical trials of PI3K and Akt inhibitors have only recently commenced. However, successful modulation of the PI3K-PTEN-Akt-mTOR pathway for anticancer therapy has been demonstrated with the mTOR inhibitor rapamycin and its analogues temsirolimus (CCI-779, Torisel; Wyeth Pharmaceuticals), everolimus (RAD0991; Novartis) and deforolimus (AP23573; ARIAD and Merck) (reviewed in [90]) While clinical efficacy has been demonstrated in a range of tumour types, including Phase II studies in patients with advanced endometrial cancer, objective response rates have been modest. Preclinical studies have shown that these mTOR inhibitors are primarily cytostatic as single agents [100], and therefore future use may be aimed at preventing disease progression or in combination with chemo-radiotherapy. Several clinical trials with mTOR inhibitors are currently underway; i) a phase II clinical trial assessing single agent temsirolimus in ovarian, fallopian tube or primary peritoneal cancer ii) a phase I study in combination with topotecan in patients with gynaecological cancers and iii) a study of temsirolimus in patients with ovarian cancer with CA125 only relapse investigating progression-free survival. It is also worthy of note that a new generation of dual mTOR inhibitors (targeting both mTOR1 and mTOR2) are currently being developed, e.g. BEZ2354 (Novartis, East Hanover, NJ), EX147 (Exelixis, San Francisco, CA), which may be more efficacious compared with first generation compounds. In addition to determining toxicity and efficacy, there is an urgent need to identify molecular markers that predict responsiveness, which thus far has been hampered by limited tissue specimens and small patient numbers achieving objective responses. Recently, in a small study of 41 women with ovarian tumours, dynamic contrast-enhanced MRI has been shown to distinguish between benign, borderline and invasive tumours and correlate with tumoural angiogenic status e.g. VEGFR-2 by immunohistochemistry, potentially negating the need for biopsy specimens and permitting more accurate response assessment [101].

## 6. Immunologic Agents

Ca-125 is a surface glycoprotein antigen elevated in more than 95% of patients with stage III and IV ovarian cancers [102]. While Ca125 is also elevated in other malignancies as well as in benign tumours, serum levels are used to monitor response to chemotherapy and predict recurrence. Oregovomab (OvaRexTM, ViRexx Medical Corp., Edmonton, Canada) is a monoclonal antibody that stimulates a humoral immune response by strongly binding to Ca-125 forming complexes that the immune system recognizes as foreign [103,104]. Two Phase II studies have demonstrated a survival advantage following the administration of oregovomab both as a single agent and in combination with cytotoxic chemotherapy [105,106]. However, a larger, randomized study in 145 patients with stage III/IV ovarian cancer patients with a complete clinical response, who received either maintenance oregovomab therapy or a placebo, failed to identify any difference in time to relapse (TTR), the primary endpoint [107]. The failure of this study is perhaps indicative of the inability of single agent oregovomab to induce a substantial immune response in the maintenance setting. The magnitude of the immune response with maintenance immunotherapy is much lower when compared to that achieved with combination chemoimmunotherapy. This was demonstrated in a front-line pilot study of oregovomab administered with carboplatin and paclitaxel [108]. Concurrent administration of antibody with chemotherapy was more immunogenic than delayed infusion supporting a new treatment paradigm that requires further investigation. 

An anti-EPCAM and anti-CD3 antibody currently undergoing clinical evaluation is catumaxomab (Removab®, TRION Pharma GmbH & Fresenius Biotech, Germany) which is known to kill tumour cells within ascitic fluid by activating T cells and accessory cells. A randomised study of catumaxomab in patients with malignant ascites (50% of which had ovarian cancer) demonstrated that patients that received intraperitoneal infusions of catumaxomab had a significant prolongation of puncture-free survival and puncture-free time [109]. Side effects were manageable and largely related to cytokine release. Ovarian cancer patients appeared to derive greater benefit than patients with malignant ascites from nonovarian carcinomas. 

Antifolate agents are also in clinical development. The folate transporter α-FR is overexpressed in 90% of ovarian cancers compared with normal tissue, possibly promoting growth by increasing folate availability [110]. The degree of overexpression appears to correlate with the grade of the tumour. Farletuzumab (MORAb-003, Morphteck Inc., PA) is a humanized IgG1 antibody to α-FR, inhibiting the growth of cells that overexpress α-FR and activating both cell- and complement-mediated cytotoxicity [111]. In a Phase II study of 54 patients with platinum-sensitive disease in first relapse, promising signs of efficacy were shown; normalization of Ca-125 levels and longer remissions for 12 patients [112]. BGC 945 (BTG, London), currently in preclinical development, is a potent thymidylate synthase inhibitor specifically transported into α-FR, and in contrast with conventional antifolates demonstrates less affinity for ubiquitously expressed folate transporter 1 [113].

## 7. Conclusions

The emergence of targeted therapies offers the potential of less (different) toxicities and improved efficacy, both as single agents based on specific molecular aberrations, and in combination with conventional cytotoxics to overcome drug resistance. One key challenge is determining the best placement of targeted therapies within the armament of existing treatments: first-line, maintenance / consolidation therapy, at relapse / remission, in combination. Traditionally, novel agents are often combined with existing cytotoxics, such as in the ICON-7 trial in which patients with newly diagnosed ovarian epithelial, fallopian tube or primary peritoneal cancer receive carboplatin and paclitaxel chemotherapy with or without bevacizumab. Likewise the ICON 6 study is investigating the efficacy of cediranib, an oral VEGF receptor tyrosine kinase inhibitor with chemotherapy in relapsed platinum-sensitive ovarian cancer. Combining targeted therapies poses the additional question of whether it is most efficacious to inhibit different signalling pathways (horizontal blockade) or different molecules within the same pathway to counteract pathway redundancy (vertical blockade). The pitfalls of combining targeted agents have been demonstrated in trials combining bevacizumab and sorafenib [51], and bevacizumab and erlotinib [53], which unexpectedly reported severe toxicities. There is also a lack of knowledge about the relevance of a molecular marker to an individual tumour, a phenomenon termed oncogene addiction [114]. For example, in colorectal cancer response to cetuximab strongly correlates with mutational status of KRAS but the assumption that this translates to other disease settings is false: EGFR mutations are not predictive of response to treatment in patients with ovarian cancer [115] while in advanced gastric cancer KRAS and BRAF mutations are significantly lower, and also do not correlate with response to cetuximab [116].

In future it is unlikely that ovarian cancer will be treated as a single entity, but will instead be based on molecular characterisation. From the evidence presented here, patients with homologous recombination-defective tumours could be considered for PARP inhibitors and endometrioid subtypes could be candidates for PI3K-PTEN-Akt-mTOR therapies. However, it is unlikely that histological subtype alone will be sufficient to account for tumour molecular heterogeneity. Molecular profiling of the tumour and normal tissues will enable better understanding of the effects of inhibiting the target in tumour and host tissue. Additionally, genetic polymorphisms may significantly affect drug distribution and tolerance and this is being harnessed in the field of pharmacogenomics. This will enable patients to be selected for a treatment to which they are most likely to respond (but may also potentially deny biomarker negative patients a treatment from which they may benefit). In order to better understand molecular aberrations in ovarian cancer, clinical trials will need to mandate the collection of high quality reference material before, during and after treatment e.g. at disease relapse. This should not detract from clinical parameters and will permit correlation with toxicity and response evaluation. In the future, the ‘diagnostic biopsy’ will be characterised and the patient ‘profiled’ to select a treatment to which the patient will most likely respond. This archived material can then be compared with tumour tissue at disease relapse, to identify stem cells that may account for a resistant population of tumour cells [117].

Traditionally, tumour biopsies have been the gold-standard reference material used to predict responsiveness to a therapy. However, difficulty in obtaining samples has led to the development of surrogate end-points and the inclusion of pharmacodynamic endpoints in clinical trials to establish proof of mechanism. Optimal development requires a reliable, validated assay to evaluate target inhibition and a knowledge of the predicted effects of target inhibition, e.g., tumour shrinkage, cytostasis, decreased vasculature. Extensive preclinical studies, both in vitro and in vivo are required to expedite drug development so that clinical trials can be targeted to patients who carry the relevant molecular aberrations. Traditional phase I end-points such as toxicity may need to be replaced in order to define the biologically effective dose with molecularly targeted anticancer agents e.g. plasma drug concentration or target inhibition in surrogate tissue. Primary end-points such as objective tumour response by anatomical imaging (assessed by Response Evaluation Criteria in Solid Tumours-RECIST) commonly used for conventional cytotoxic drugs may not detect cytostatic effects and functional imaging such as DCE-MRI and FDG-PET may be more useful biomarkers. The co-development of novel targeted drugs with biomarkers is essential as new treatments are associated with high costs, and many fail unpredictably in late clinical trials (reviewed in [118]). Comparatively, the costs of biomarker trials are low (typically <15% of the cost of the clinical trials), and are essential in an era where drug approvals are falling while research and development costs increase.

## References

[1] Parkin D.M., Bray F., Ferlay J., Pisani P. (2005). Global cancer statistics, 2002. CA Cancer J. Clin..

[2] Jemal A., Siegel R., Ward E., Hao Y., Xu J., Murray T., Thun M.J. (2008). Cancer statistics, 2008. CA Cancer J. Clin..

[3] Dinh P., Harnett P., Piccart-Gebhart M.J., Awada A. (2008). New therapies for ovarian cancer: cytotoxics and molecularly targeted agents. Crit. Rev. Oncol. Hematol..

[4] Greenlee R.T., Hill-Harmon M.B., Murray T., Thun M. (2001). Cancer statistics, 2001. CA Cancer J. Clin..

[5] Ozols R.F., Bundy B.N., Greer B.E., Fowler J.M., Clarke-Pearson D., Burger R.A., Mannel R.S., DeGeest K., Hartenbach E.M, Baergen R. (2003). Phase III trial of carboplatin and paclitaxel compared with cisplatin and paclitaxel in patients with optimally resected stage III ovarian cancer: a Gynecologic Oncology Group study. J. Clin. Oncol..

[6] Biagi J.J., Eisenhauer E.A. (2003). Systemic treatment policies in ovarian cancer: the next 10 years. Int. J. Gynecol. Cancer.

[7] Sandercock J., Parmar M.K., Torri V., Qian W. (2002). First-line treatment for advanced ovarian cancer: paclitaxel, platinum and the evidence. Br. J. Cancer.

[8] Cannistra S.A. (2004). Cancer of the ovary. N. Engl. J. Med..

[9] Agarwal R., Kaye S.B. (2003). Ovarian cancer: strategies for overcoming resistance to chemotherapy. Nat. Rev. Cancer.

[10] Demetri G.D., von Mehren M., Blanke C.D., van den Abbeele A.D., Eisenberg B., Roberts P.J., Heinrich M.C., Tuveson D.A., Singer S., Janicek M. (2002). Efficacy and safety of imatinib mesylate in advanced gastrointestinal stromal tumors. N. Engl. J. Med..

[11] Hurwitz H., Fehrenbacher L., Novotny W., Cartwright T., Hainsworth J., Heim W., Berlin J., Baron A., Griffing S., Holmgren E. (2004). Bevacizumab plus irinotecan, fluorouracil, and leucovorin for metastatic colorectal cancer. N. Engl. J. Med..

[12] Zondor S.D., Medina P.J. (2004). Bevacizumab: an angiogenesis inhibitor with efficacy in colorectal and other malignancies. Ann. Pharmacother..

[13] Miller K., Wang M., Gralow J., Dickler M., Cobleigh M., Perez E.A., Shenkier T., Cella D., Davidson N.E. (2007). Paclitaxel plus Bevacizumab versus Paclitaxel Alone for Metastatic Breast Cancer. N. Engl. J. Med..

[14] Sandler A., Herbst R. (2006). Combining Targeted Agents: Blocking the Epidermal Growth Factor and Vascular Endothelial Growth Factor Pathways. Clin. Cancer Res..

[15] Llovet J., Ricci S., Mazzaferro V., Hilgard P., Raoul J., Zeuzern S., Poulin-Costello M., Moscovici M., Voliotis D., Bruix J. (2007). Sorafenib improves survival in advanced Hepatocellular Carcinoma (HCC): Result of a Phase III randomised placebo-controlled trial (SHARP trial). J. Clin. Oncol..

[16] Escudier B., Eisen T., Stadler W.M., Szczylik C., Oudard S., Siebels M., Negrier S., Chevreau C., Solska E., Desai A.A. (2007). Sorafenib in advanced clear-cell renal-cell carcinoma. N. Engl. J. Med..

[17] Motzer R.J., Hutson T.E., Tomczak P., Michaelson M.D., Bukowski R.M., Rixe O., Oudard S., Negrier S., Szczylik C., Kim S.T. (2007). Sunitinib versus interferon alfa in metastatic renal-cell carcinoma. N. Engl. J. Med..

[18] Martin L., Schilder R. (2007). Novel approaches in advancing the treatment of epithelial ovarian cancer: the role of angiogenesis inhibition. J. Clin. Oncol..

[19] Stone P.J., Goodheart M.J., Rose S.L., DeYoung B.R., Buller R.E. (2003). The influence of microvessel density on ovarian carcinogenesis. Gynecol. Oncol..

[20] Goodheart M.J., Ritchie J.M., Rose S.L., Fruehauf J.P., DeYoung B.R., Buller R.E. (2005). The relationship of molecular markers of p53 function and angiogenesis to prognosis of stage I epithelial ovarian cancer. Clin. Cancer Res..

[21] Hefler L.A., Mustea A., Konsgen D., Concin N., Tanner B., Strick R., Heinze G., Grimm C., Schuster E., Tempfer C. (2007). Vascular endothelial growth factor gene polymorphisms are associated with prognosis in ovarian cancer. Clin. Cancer Res..

[22] Byrne A.T., Ross L., Holash J., Nakanishi M., Hu L., Hofmann J.I., Yancopoulos G.D., Jaffe R.B. (2003). Vascular endothelial growth factor-trap decreases tumor burden, inhibits ascites, and causes dramatic vascular remodeling in an ovarian cancer model. Clin. Cancer Res..

[23] Schumacher J.J., Dings R.P., Cosin J., Subramanian I.V., Auersperg N., Ramikrishnan S. (2007). Modulation of angiogenic phenotype alters tumorigenicity in rat ovarian epithelial cells. Cancer Res..

[24] Spannuth W.A., Sood A.K., Coleman R.L. (2008). Angiogenesis as a strategic target for ovarian cancer therapy. Nat. Clin. Pract. Oncol..

[25] Rosa D.D., Clamp A.R., Collinson F., Jayson G.C. (2007). Antiangiogenic therapy for ovarian cancer. Curr. Opin. Oncol..

[26] Mesiano S., Ferrara N., Jaffe R.B. (1998). Role of vascular endothelial growth factor in ovarian cancer: inhibition of ascites formation by immunoneutralization. Am. J. Pathol..

[27] Hu L., Hofmann J., Lu Y., Mills G.B., Jaffe R.B. (2002). Inhibition of phosphatidylinositol 3'-kinase increases efficacy of paclitaxel in vitro and in vivo ovarian cancer models. Cancer Res..

[28] Mabuchi S., Terai Y., Morishige K., Tanabe-Kimura A., Sasaki H., Kanemura M., Tsunetoh S., Tanaka Y., Sakata M., Burger R.A. (2008). Maintenance treatment with bevacizumab prolongs survival in an in vivo ovarian cancer model. Clin. Cancer Res..

[29] Cannistra S.A., Matulonis U.A., Penson R.T., Hambleton J., Dupont J., Mackey H., Douglas J., Burger R.A., Armstrong D., Wenham R. (2007). Phase II study of bevacizumab in patients with platinum-resistant ovarian cancer or peritoneal serous cancer. J. Clin. Oncol..

[30] Numnum T.M., Rocconi R.P., Whitworth J., Barnes M.N. (2006). The use of bevacizumab to palliate symptomatic ascites in patients with refractory ovarian carcinoma. Gynecol. Oncol..

[31] Monk B.J., Han E., Josephs-Cowan C.A., Pugmire G., Burger R.A. (2006). Salvage bevacizumab (rhuMAB VEGF)-based therapy after multiple prior cytotoxic regimens in advanced refractory epithelial ovarian cancer. Gynecol. Oncol..

[32] Burger R.A., Sill M.W., Monk B.J., Greer B.E., Sorosky J.I. (2007). Phase II trial of bevacizumab in persistent or recurrent epithelial ovarian cancer or primary peritoneal cancer: a Gynecologic Oncology Group Study. J. Clin. Oncol..

[33] Garcia A.A., Hirte H., Fleming G., Yang D., Tsao-Wei D.D., Roman L., Groshen S., Swenson S., Markland F., Gandara D. (2008). Phase II clinical trial of bevacizumab and low-dose metronomic oral cyclophosphamide in recurrent ovarian cancer: a trial of the California, Chicago, and Princess Margaret Hospital phase II consortia. J. Clin. Oncol..

[34] Wright J.D., Secord A.A., Numnum T.M., Rocconi R.P., Powell M.A., Berchuck A., Alvarez R.D., Gibb R.K., Trinkaus K., Rader J.S. (2008). A multi-institutional evaluation of factors predictive of toxicity and efficacy of bevacizumab for recurrent ovarian cancer. Int. J. Gynecol. Cancer.

[35] Fukumura D., Jain R.K. (2007). Tumor microenvironment abnormalities: causes, consequences, and strategies to normalize. J. Cell Biochem..

[36] Hanahan D., Bergers G., Bergsland E. (2000). Less is more, regularly: metronomic dosing of cytotoxic drugs can target tumor angiogenesis in mice. J. Clin. Invest..

[37] Klement G., Baruchel S., Rak J., Man S., Clark K., Hicklin D.J., Bohlen P., Kerbel R.S. (2000). Continuous low-dose therapy with vinblastine and VEGF receptor-2 antibody induces sustained tumor regression without overt toxicity. J. Clin. Invest..

[38] Kamat A.A., Kim T.J., Landen C.N., Lu C., Han L.Y., Lin Y.G., Merritt W.M., Thaker P.H., Gershenson D.M., Bischoff F.Z. (2007). Metronomic chemotherapy enhances the efficacy of antivascular therapy in ovarian cancer. Cancer Res..

[39] Chura J.C., van Iseghem K., Downs L.S., Carson L.F., Judson P.L. (2007). Bevacizumab plus cyclophosphamide in heavily pretreated patients with recurrent ovarian cancer. Gynecol. Oncol..

[40] Micha J.P., Goldstein B.H., Rettenmaier M.A., Genesen M., Graham C., Bader K., Lopez K.L., Nickle M., Brown J.V. (2007). A phase II study of outpatient first-line paclitaxel, carboplatin, and bevacizumab for advanced-stage epithelial ovarian, peritoneal, and fallopian tube cancer. Int. J. Gynecol. Cancer.

[41] Campos S.M., Dizon D.S., Cannistra S.A., Roche M., Krasner C.N., Berlin S.T., Horowitz N.S., DiSilvestro P., Matulonis U.A., Penson R.T. (2007). Safety of maintenance bevacizumab after first-line chemotherapy for advanced ovarian and müllerian cancers. J. Clin. Oncol..

[42] Kabbinavar F., Hurwitz H.I., Fehrenbacher L., Meropol N.J., Novotny W.F., Lieberman G., Griffing S., Bergsland E. (2003). Phase II, Randomized Trial Comparing Bevacizumab Plus Fluorouracil (FU)/Leucovorin (LV) With FU/LV Alone in Patients With Metastatic Colorectal Cancer. J. Clin. Oncol..

[43] Tew W.P., Colombo N., Ray-Coquard I., Oza A., del Campo J., Scambia G., Spriggs D. (2007). VEGF-Trap for patients (pts) with recurrent platinum-resistant epithelial ovarian cancer (EOC): Preliminary results of a randomized, multicenter phase II study. J. Clin. Oncol..

[44] Biagi J.J., Oza A.M., Grimshaw R., Ellard S.L., Lee U., Sederias J., Ivy S.P., Eisenhauer E.A. (2008). A phase II study of sunitinib (SU11248) in patients (pts) with recurrent epithelial ovarian, fallopian tube or primary peritoneal carcinoma - NCIC CTG IND 185. J. Clin. Oncol..

[45] Matulonis U.A., Berlin S.T., Krasner C.N., Tyburski K., Lee J., Roche M., Ivy S.P., Lenahan C., King M., Penson R.T. (2008). Cediranib (AZD2171) is an active agent in recurrent epithelial ovarian cancer. J. Clin. Oncol..

[46] Matei D., Sill M.W., DeGeest K., Bristow R.E. (2008). Phase II trial of sorafenib in persistent or recurrent epithelial ovarian cancer (EOC) or primary peritoneal cancer (PPC): A Gynecologic Oncology Group (GOG) study. J. Clin. Oncol..

[47] Friedlander M., Hancock K.C., Benigno B., Rischin D., Messing M., Stringer C.A., Tay E.H., Kathman S., Matthys G., Lager J.J. (2007). Pazopanib (GW786034) is active in women with advanced epithelial ovarian, fallopian tube and peritoneal cancers: Initial results of a phase II study. J. Clin. Oncol..

[48] Coleman R.L., Broaddus R.R., Bodurka D.C., Wolf J.K., Burke T.W., Kavanagh J.J., Levenback C.F., Gershenson D.M. (2006). Phase II trial of imatinib mesylate in patients with recurrent platinum- and taxane-resistant epithelial ovarian and primary peritoneal cancers. Gynecol. Oncol..

[49] Posadas E.M., Kwitkowski V., Kotz H.L., Espina V., Minasian L., Tchabo N., Premkumar A., Hussain M.M., Chang R., Steinberg S.M. (2007). A prospective analysis of imatinib-induced c-KIT modulation in ovarian cancer: a phase II clinical study with proteomic profiling. Cancer.

[50] Hirte H.W., Vidal L., Fleming G.F., Sugimoto A.K., Morgan R.J., Biagi J.J., Wang L., McGill S., Ivy S.P., Oza A.M. (2008). A phase II study of cediranib (AZD2171) in recurrent or persistent ovarian, peritoneal or fallopian tube cancer: Final results of a PMH, Chicago and California consortia trial. J. Clin. Oncol..

[51] Azad N.S., Posadas E.M., Kwitkowski V.E., Steinberg S.M., Jain L., Annunziata C.M., Minasian L., Sarosy G., Kotz H.L., Premkumar A. (2008). Combination targeted therapy with sorafenib and bevacizumab results in enhanced toxicity and antitumor activity. J. Clin. Oncol..

[52] Sandler A., Herbst R. (2006). Combining targeted agents: blocking the epidermal growth factor and vascular endothelial growth factor pathways. Clin. Cancer Res..

[53] Nimeiri H.S., Oza A.M., Morgan R.J., Friberg G., Kasza K., Faoro L., Salgia R., Stadler W.M., Vokes E.E., Fleming G.F. (2008). Efficacy and safety of bevacizumab plus erlotinib for patients with recurrent ovarian, primary peritoneal, and fallopian tube cancer: a trial of the Chicago, PMH, and California Phase II Consortia. Gynecol. Oncol..

[54] Hecht J.R., Mitchell E., Chidiac T., Scroggin C., Hagenstad C., Spigel D., Marshall J., Cohn A., McCollum D., Stella P (2009). A randomized phase IIIB trial of chemotherapy, bevacizumab, and panitumumab compared with chemotherapy and bevacizumab alone for metastatic colorectal cancer. J. Clin. Oncol..

[55] Nathan P.D., Judson I., Padhani A., Harris A., Carden C.P., Smythe J., Collins D., Leach M., Walicke P., Rustin G.J. (2008). A phase I study of combretastatin A4 phosphate (CA4P) and bevacizumab in subjects with advanced solid tumors. J. Clin. Oncol..

[56] Shaked Y., Ciarrocchi A., Franco M., Lee C.R., Man S., Cheung A.M., Hicklin D.J., Chaplin D., Foster F.S., Benezra R. (2006). Therapy-induced acute recruitment of circulating endothelial progenitor cells to tumors. Science.

[57] Gridelli C., Rossi A., Maione P., Rossi E., Castaldo V., Sacco P.C., Colantuoni G. (2009). Vascular disrupting agents: a novel mechanism of action in the battle against non-small cell lung cancer. Oncologist.

[58] Zweifel M., Jayson G., Reed N., Osborne R., Hassan B., Shrreves G., Poupard L., Walicke A., Balkissoon J., Chaplin D. (2009). Combretastatin A-4 phosphate (CA4P) carboplatin and paclitaxel in patients with platinum-resistant ovarian cancer: Final phase II trial results. J. Clin. Oncol..

[59] Hapani S., Chu D., Wu S. (2009). Risk of gastrointestinal perforation in patients with cancer treated with bevacizumab: a meta-analysis. Lancet Oncol..

[60] Han E.S., Monk B.J. (2007). What is the risk of bowel perforation associated with bevacizumab therapy in ovarian cancer?. Gynecol. Oncol..

[61] Zhu X., Wu S., Dahut W.L., Parikh C.R. (2007). Risks of proteinuria and hypertension with bevacizumab, an antibody against vascular endothelial growth factor: Systematic review and meta-analysis. Am. J. Kidney Dis..

[62] Yang J.C., Haworth L., Sherry R.M., Hwu P., Schwartzentruber D.J., Topalian S.L., Steinberg S.M., Chen H.X., Rosenberg S.A. (2003). A randomized trial of bevacizumab, an anti-vascular endothelial growth factor antibody, for metastatic renal cancer. N. Engl. J. Med..

[63] Veronese M.L., Mosenkis A., Flaherty K.T., Gallagher M., Stevenson J.P., Townsend R.R., O'Dwyer P.J. (2006). Mechanisms of hypertension associated with BAY 43-9006. J. Clin. Oncol..

[64] Alper O., Bergmann-Leitner E.S., Bennett T.A., Hacker N.F., Stromberg K., Stetler-Stevenson W.G. (2001). Epidermal growth factor receptor signaling and the invasive phenotype of ovarian carcinoma cells. J. Nat. Cancer Inst..

[65] Skirnisdottir I., Sorbe B., Seidal T. (2001). The growth factor receptors HER-2/neu and EGFR, their relationship, and their effects on the prognosis in early stage (FIGO I-II) epithelial ovarian carcinoma. Int. J. Gynecol. Cancer.

[66] Mendelsohn J., Baselga J. (2003). Status of epidermal growth factor receptor antagonists in the biology and treatment of cancer. J. Clin. Oncol..

[67] Goldstein N.I., Prewett M., Zuklys K., Rockwell P., Mendelsohn J. (1995). Biological efficacy of a chimeric antibody to the epidermal growth factor receptor in a human tumor xenograft model. Clin. Cancer Res..

[68] Seiden M.V., Burris H.A., Matulonis U., Hall J.B., Armstrong D.K., Speyer J., Weber J.D., Muggia F. (2007). A phase II trial of EMD72000 (matuzumab), a humanized anti-EGFR monoclonal antibody, in patients with platinum-resistant ovarian and primary peritoneal malignancies. Gynecol. Oncol..

[69] Calvo E., Tolcher A.W., Hammond L.A., Patnaik A., de Bono J.S., Eiseman I.A., Olson S.C., Lenehan P.F., McCreery H., Lorusso P. (2004). Administration of CI-1033, an Irreversible Pan-erbB Tyrosine Kinase Inhibitor, Is Feasible on a 7-Day On, 7-Day Off Schedule. Clin. Cancer Res..

[70] Finkler N., Gordon A., Crozier M., Edwards R., Figueroa J., Garcia A., Hainsworth J., Irwin D., Silberman S., Allen L. (2001). Phase 2 Evaluation of OSI-774, a Potent Oral Antagonist of the EGFR-TK in Patients with Advanced Ovarian Carcinoma. J. Clin. Oncol..

[71] Aghajanian C., Sabbatini P., Derosa F., Gerst S., Spriggs D.R., Dupont J., Hensley M.L., Pezzulli S., Konner J., Schilder R.J. (2005). A Phase II Study of Cetuximab/Paclitaxel/Carboplatin for the Initial Treatment of Advanced Stage Ovarian, Primary Peritoneal, and Fallopian Tube Cancer. J. Clin. Oncol..

[72] Amado R.G., Wolf M., Peeters M., van Cutsem E., Siena S., Freeman D.J., Juan T., Sikorski R., Suggs S., Radinsky R. (2008). Wild-type KRAS is required for panitumumab efficacy in patients with metastatic colorectal cancer. J. Clin. Oncol..

[73] Shih I.M., Kurman R.J. (2004). Ovarian tumorigenesis: a proposed model based on morphological and molecular genetic analysis. Am. J. Pathol.

[74] Schilder R.J., Sill M.W., Chen X., Darcy K.M., Decesare S.L., Lewandowski G., Lee R.B., Arciero C.A., Wu H., Godwin A.K. (2005). Phase II study of gefitinib in patients with relapsed or persistent ovarian or primary peritoneal carcinoma and evaluation of epidermal growth factor receptor mutations and immunohistochemical expression: a Gynecologic Oncology Group Study. Clin. Cancer Res..

[75] Bookman M.A., Darcy K.M., Clarke-Pearson D., Boothby R.A., Horowitz I.R. (2003). Evaluation of monoclonal humanized anti-HER2 antibody, trastuzumab, in patients with recurrent or refractory ovarian or primary peritoneal carcinoma with overexpression of HER2: a phase II trial of the Gynecologic Oncology Group. J. Clin. Oncol..

[76] Gordon M.S., Matei D., Aghajanian C., Matulonis U.A., Brewer M., Fleming G.F., Hainsworth J.D., Garcia A.A., Pegram M.D., Schilder R.J. (2006). Clinical activity of pertuzumab (rhuMAb 2C4), a HER dimerization inhibitor, in advanced ovarian cancer: potential predictive relationship with tumor HER2 activation status. J. Clin. Oncol..

[77] Amler L., Makhija S., Januario T., Matulonis U.A., Strauss A., Dizon D.S., Sliwkowski M.X., Dolezal M., Tong B., Paton V. (2008). HER pathway gene expression analysis in a phase II study of pertuzumab + gemcitabine *vs.* gemcitabine + placebo in patients with platinum-resistant epithelial ovarian cancer. J. Clin. Oncol..

[78] Schreiber V., Dantzer F., Ame J.C., de Murcia G. (2006). Poly(ADP-ribose): novel functions for an old molecule. Nat Rev Mol Cell Biol.

[79] Ame J.C., Spenlehauer C., de Murcia G. (2004). The PARP superfamily. Bioessays.

[80] Bryant H.E., Schultz N., Thomas H.D., Parker K.M., Flower D., Lopez E., Kyle S., Meuth M., Curtin N.J., Helleday T. (2005). Specific killing of BRCA2-deficient tumours with inhibitors of poly(ADP-ribose) polymerase. Nature.

[81] Farmer H., McCabe N., Lord C.J., Tutt A.N., Johnson D.A., Richardson T.B., Santarosa M., Dillon K.J., Hickson I., Knights C. (2005). Targeting the DNA repair defect in BRCA mutant cells as a therapeutic strategy. Nature.

[82] Hay T., Jenkins H., Sansom O.J., Martin N.M., Smith G.C., Clarke A.R. (2005). Efficient deletion of normal Brca2-deficient intestinal epithelium by poly(ADP-ribose) polymerase inhibition models potential prophylactic therapy. Cancer Res..

[83] Fong P.C., Spicer J., Reade S., Reid A., Vidal L., Schellens J.H., Tutt A., Harris P.A., Kaye S., DeBono J.S. (2006). Phase I pharmacokinetic (PK) and pharmacodynamic (PD) evaluation of a small molecule inhibitor of Poly ADP-Ribose Polymerase (PARP), KU-0059436 (Ku) in patients (p) with advanced tumours. J. Clin. Oncol..

[84] Tutt A.N., Lord C.J., McCabe N., Farmer H., Turner N., Martin N.M., Jackson S.P., Smith G.C., Ashworth A. (2005). Exploiting the DNA repair defect in BRCA mutant cells in the design of new therapeutic strategies for cancer. Cold Spring Harb. Symp. Quant. Biol..

[85] Fong P.C., Boss D.S., Carden C.P., Roelvink M., De Greve J., Gourley C.M., Carmichael J., De Bono J.S., Schellens J.H., Kaye S.B. (2008). AZD2281 (KU-0059436), a PARP (poly ADP-ribose polymerase) inhibitor with single agent anticancer activity in patients with BRCA deficient ovarian cancer: Results from a phase I study. J. Clin. Oncol..

[86] Audeh M.W., Penson R.T., Friedlander M., Powell B., Bell-McGuinn K.M., Scott C., Weitzel J.N., Carmichael J., Tutt A. (2009). Phase II trial of the oral PARP inhibitor olaparib (AZD2281) in BRCA-deficient advanced ovarian cancer. J. Clin. Oncol..

[87] Turner N., Tutt A., Ashworth A. (2004). Hallmarks of 'BRCAness' in sporadic cancers. Nat. Rev. Cancer.

[88] Press J.Z., De Luca A., Boyd N., Young S., Troussard A., Ridge Y., Kaurah P., Kalloger S.E., Blood K.A., Smith M. (2008). Ovarian carcinomas with genetic and epigenetic BRCA1 loss have distinct molecular abnormalities. BMC Cancer.

[89] Hennessy B.T., Smith D.L., Ram P.T., Lu Y., Mills G.B. (2005). Exploiting the PI3K/AKT pathway for cancer drug discovery. Nat. Rev. Drug Discov..

[90] Meric-Bernstam F., Gonzalez-Angulo A.M. (2009). Targeting the mTOR signaling network for cancer therapy. J. Clin. Oncol..

[91] Shayesteh L., Lu Y., Kuo W.L., Baldocchi R., Godfrey T., Collins C., Pinkel D., Powell B., Mills G.B., Gray J.W. (1999). PIK3CA is implicated as an oncogene in ovarian cancer. Nat. Genet..

[92] Campbell I.G., Russell S.E., Choong D.Y., Montgomery K.G., Ciavarella M.L., Hooi C.S., Cristiano B.E., Pearson R.B., Phillips W.A. (2004). Mutation of the PIK3CA gene in ovarian and breast cancer. Cancer Res..

[93] Obata K., Morland S.J., Watson R.H., Hitchcock A., Chenevix-Trench G., Thomas E.J., Campbell I.G. (1998). Frequent PTEN/MMAC mutations in endometrioid but not serous or mucinous epithelial ovarian tumors. Cancer Res..

[94] Wolf J., Slomovitz B.M. (2005). Novel biologic terapies for the treatment of endometrial cancer. Int. J. Gynecol. Cancer.

[95] Hay N. (2005). The Akt-mTOR tango and its relevance to cancer. Cancer Cell.

[96] Altomare D.A., Wang H.Q., Skele K.L., De Rienzo A., Klein-Szanto A.J., Godwin A.K., Testa J.R. (2004). AKT and mTOR phosphorylation is frequently detected in ovarian cancer and can be targeted to disrupt ovarian tumor cell growth. Oncogene.

[97] Trinh X.B., Tjalma W.A., Vermeulen P.B., van den Eynden G., van der Auwera I., van Laere S.J., Helleman J., Berns E.M., Dirix L.Y., van Dam P.A. (2009). The VEGF pathway and the AKT/mTOR/p70S6K1 signalling pathway in human epithelial ovarian cancer. Br. J. Cancer.

[98] Xing H., Weng D., Chen G., Tao W., Zhu T., Yang X., Meng L., Wang S., Lu Y., Ma D. (2008). Activation of fibronectin/PI-3K/Akt2 leads to chemoresistance to docetaxel by regulating survivin protein expression in ovarian and breast cancer cells. Cancer Lett..

[99] Fraser M., Bai T., Tsang B.K. (2008). Akt promotes cisplatin resistance in human ovarian cancer cells through inhibition of p53 phosphorylation and nuclear function. Int. J. Cancer.

[100] Mondesire W.H., Jian W., Zhang H., Ensor J., Hung M.C., Mills G.B., Meric-Bernstam F. (2004). Targeting mammalian target of rapamycin synergistically enhances chemotherapy-induced cytotoxicity in breast cancer cells. Clin. Cancer Res..

[101] Thomassin-Naggara I., Darai E., Cuenod C.A., Rouzier R., Callard P., Bazot M. (2008). Dynamic contrast-enhanced magnetic resonance imaging: a useful tool for characterizing ovarian epithelial tumors. J. Magn. Reson. Imaging.

[102] Bast R.C., Klug T.L., St John E., Jenison E., Niloff J.M., Lazarus H., Berkowitz R.S., Leavitt T., Griffiths C.T., Parker L. (1983). A radioimmunoassay using a monoclonal antibody to monitor the course of epithelial ovarian cancer. N. Engl. J. Med..

[103] Berek J.S., Schultes B.C., Nicodemus C.F. (2003). Biologic and immunologic therapies for ovarian cancer. J. Clin. Oncol..

[104] Madiyalakan R., Sykes T.R., Dharampaul S., Sykes C.J., Baum R.P., Hör G., Noujaim A.A. (1995). Antiidiotype induction therapy: evidence for the induction of immune response through the idiotype network in patients with ovarian cancer after administration of anti-CA125 murine monoclonal antibody B43.13. Hybridoma.

[105] Ehlen T.G., Hoskins P.J., Miller D., Whiteside T.L., Nicodemus C.F., Schultes B.C., Swenerton K.D. (2005). A pilot phase 2 study of oregovomab murine monoclonal antibody to CA125 as an immunotherapeutic agent for recurrent ovarian cancer. Int. J. Gynecol.Cancer.

[106] Gordon A.N., Schultes B.C., Gallion H., Edwards R., Whiteside T.L., Cermak J.M., Nicodemus C.F. (2004). CA125- and tumor-specific T-cell responses correlate with prolonged survival in oregovomab-treated recurrent ovarian cancer patients. Gynecol. Oncol.

[107] Berek J.S., Taylor P.T., Gordon A., Cunningham M.J., Finkler N., Orr J., Rivkin S., Schultes B.C., Whiteside T.L., Nicodemus C.F. (2004). Randomized, placebo-controlled study of oregovomab for consolidation of clinical remission in patients with advanced ovarian cancer. J. Clin. Oncol..

[108] Braly P., Chu C., Collins Y., Edwards R., Gordon A., McGuire W., Smith L.M., Nicodemus C., Method M. (2007). Prospective evaluation of front-line chemo-immunotherapy (C-IT) with oregovomab (2 alternative dosing schedules) carboplatin-paclitaxel (C-P) in advanced ovarian cancer (OC). J. Clin. Oncol..

[109] Parsons S., Murawa P.X., Koralewski P., Kutarska E., Kolesnik O.O., Stroehlein M.A., Lahr A., Jaeger M., Heiss M.M. (2008). Intraperitoneal treatment of malignant ascites due to epithelial tumors with catumaxomab, A phase II/III study. J. Clin. Oncol..

[110] Elnakat H., Ratnam M. (2006). Role of folate receptor genes in reproduction and related cancers. Front. Biosci..

[111] Ebel W., Routhier E.L., Foley B., Jacob S., McDonough J.M., Patel R.K., Turchin H.A., Chao Q., Kline J.B., Old L.J. (2007). Preclinical evaluation of MORAb-003, a humanized monoclonal antibody antagonizing folate receptor-alpha. Cancer Immun..

[112] Armstrong D.K., Bicher A., Coleman R.L., Gibbon D.G., Glenn D., Old L., Senzer N.N., Schneeweiss A., Verheijen R.H., White A.J., Weil S. (2008). Exploratory phase II efficacy study of MORAb-003, a monoclonal antibody against folate receptor alpha, in platinum-sensitive ovarian cancer in first relapse. J. Clin. Oncol..

[113] Gibbs D.D., Theti D.S., Wood N., Green M., Raynaud F., Valenti M., Forster M.D., Mitchell F., Bavetsias V., Henderson E. (2005). BGC 945, a novel tumor-selective thymidylate synthase inhibitor targeted to alpha-folate receptor-overexpressing tumors. Cancer Res..

[114] Weinstein I.B., Joe A. (2008). Oncogene addiction. Cancer Res..

[115] Lacroix L., Pautier P., Duvillard P., Motté N., Saulnier P., Bidart J.M., Soria J.C. (2006). Response of ovarian carcinomas to gefitinib-carboplatin-paclitaxel combination is not associated with EGFR kinase domain somatic mutations. Int. J. Cancer.

[116] Stella G., Rojas Llimpe F.L., Barone C., Falcone A., Di Fabio F., Lamba S., Martoni A.A., Siena S., Bardelli A., Pinto C. KRAS and BRAF mutational status and response to cetuximab combination therapy in advanced gastric cancer (GC) patients. 2009 Gastrointestinal Cancers Symposium.

[117] Zhang S., Balch C., Chan M.W., Lai H.C., Matei D., Schilder J.M., Yan P.S., Huang T.H., Nephew K.P. (2008). Identification and characterization of ovarian cancer-initiating cells from primary human tumors. Cancer Res..

[118] Marrer E., Dieterle F. (2008). Biomarkers in oncology drug development: rescuers or troublemakers?. Expert Opin. Drug Metab. Toxicol..

[119] Agus D.B., Gordon M.S., Taylor C., Natale R.B., Karlan B., Mendelson D.S., Press M.F., Allison D.E., Sliwkowski M.X., Lieberman G. (2005). Phase I clinical study of pertuzumab, a novel HER dimerization inhibitor, in patients with advanced cancer. J. Clin. Oncol..

[120] Vasey P., Kaye S., Paul J., Rustin G., Wilson R., Guastalla J.P., Pujade-Lauraine E., Gore M., Gabra H., Carty K. (2004). A phase Ib trial of erlotinib (E) in combination with docetaxel (D) and carboplatin (C) in untreated ovarian, fallopian tube and primary peritoneal cancers. J. Clin. Oncol..

[121] Blank S.V., Curtin J.P., Goldman N.A., Runowicz C.D., Speyer J.L., Tiersten A.D., Dancey J., Wadler S., Muggia F.M. (2006). Report of first-stage accrual for NCI 5886, a phase II study of erlotinib, carboplatin and paclitaxel as first-line treatment of ovarian cancer. J. Clin. Oncol..

[122] Slomovitz B.M., Coleman R.L., Levenback C., Jung M., Gershenson D.M., Wolf J. (2006). Phase I study of weekly topotecan and gefitinib in patients with platinum-resistant ovarian, peritoneal, or fallopian tube cancer. J. Clin. Oncol..

[123] Hariprasad R., Kumar L., Patnaik R., Gupta A., Kumar S. (2006). Maintenance therapy in epithelial ovarian cancer (EOC): Could EGFR inhibitor- gefitinib be a candidate drug? A pilot study. J. Clin. Oncol..

[124] Mavroudis D., Efstathiou E., Polyzos A., Athanasiadis A., Milaki G., Kastritis E., Kalykaki A., Saridaki Z., Dimopoulos A., Georgoulias V. (2004). A phase I-II trial of gefitinib in combination with vinorelbine and oxaliplatin as salvage therapy in women with advanced ovarian cancer (AOC). J. Clin. Oncol..

[125] Krasner C.N., Debernardo R.L., Findley M., Penson R., Matulonis U., Atkinson T., Roche M., Seiden M.V. (2005). Phase II trial of anastrazole in combination with gefitinib in women with asymptomatic mullerian cancer. J. Clin. Oncol..

[126] Minami H., Nakagawa K., Kawada K., Mukai H., Tahara M., Kurata T., Uejima H., Nogami T., Sasaki Y., Fukuoka M. (2004). A phase I study of GW572016 in patients with solid tumors. J. Clin. Oncol..

[127] Campos S.M., Seiden M.V., Oza A., Plante M., Potkul R., Hamid O., Lenehan P., Kaldjian E., Jordan C., Hirte H. (2004). A phase 2, single agent study of CI-1033 administered at two doses in ovarian cancer patients who failed platinum therapy. J. Clin. Oncol..

